# The Versatility of Extraoral Vertical Ramus Osteotomy for Mandibular Prognathism: A Prospective Study

**DOI:** 10.7759/cureus.32673

**Published:** 2022-12-18

**Authors:** Subalakshmi Krishnamurthy, Saravanan Balasubramaniam, Appadurai Rajenthiran, Rohini Thirunavukkarasu

**Affiliations:** 1 Oral and Maxillofacial Surgery, Thiruvannamalai Medical College and Hospital, Thiruvannamalai, IND; 2 Oral and Maxillofacial Surgery, Tamil Nadu Government Dental College and Hospital, Chennai, IND; 3 Oral and Maxillofacial Surgery, Dr. M.G.R. Medical University, Chennai, IND

**Keywords:** vertical ramus osteotomy, antilingula, orthognathic surgery, extraoral vertical ramus osteotomy, mandibular prognathism

## Abstract

Introduction: Orthognathic surgery simply means alignment of the jaws. The aim of orthognathic surgery is to normalize the relationship of the jaws between themselves and the rest of the craniofacial complex. Mandibular prognathism is a common clinical problem all over the world. Currently, sagittal ramus osteotomy is the primary choice for correcting most cases of mandibular retrognathism and prognathism. The surgical option for extreme cases of mandibular prognathism is extraoral vertical ramus osteotomy (EVRO) or intraoral vertical ramus osteotomy (IVRO) or inverted L osteotomy.

Aim: The aim of this study was to evaluate the versatility of EVRO for mandibular prognathism.

Materials and methods: Ten patients with the chief complaint of mandibular prognathism with no history of keloid tendency were included in the study. EVRO was done for all patients. The parameters based on which the outcome of the surgical procedure was assessed were time taken for the surgical procedure, facial harmony both in frontal and profile views postoperatively, and intraoperative and postoperative complications, and assessment of the postoperative results was done through orthopantomogram (OPG) and 3D CT scan.

Results: The time taken for the entire surgical procedure, starting from incision to closure, varied between 80 and 94 minutes with the average time taken for the surgery being 90 ± 8.80 minutes. It was found that there was a statistically significant difference between preoperative (M = 53.4, SD = 5.854) and postoperative evaluation (M = 47.5, SD = 5.039) of the posterior nasal spine to nasion 1 (PNS-N 1) horizontal plane (HP) (mm) with p < 0.001. Similarly, there was a statistically significant difference between preoperative (M = 81.4, SD = 2.716) and postoperative evaluation (M = 74.4, SD = 3.627) of mandible body length (mm) with p < 0.001. However, no statistically significant difference exists between the preoperative and postoperative evaluation of anterior nasal spine (ANS) to PNS (mm) and ramus height Ar-Go (mm).

Conclusion: EVRO is an acceptable surgical procedure owing to the fact that it is relatively simple to carry out, its lack of complications, and its good results.

## Introduction

Orthognathic surgery involves the surgical correction of the deformities or malpositioning of the facial skeleton. The aim of orthognathic surgery is to normalize the relationship between the jaws and the rest of the craniofacial complex [1]. Orthognathic surgery can be used to manage a broad spectrum of maxillofacial abnormalities including congenital, developmental, and acquired deformities [2]. Correction of maxillofacial deformities requires careful analysis of the soft tissue with clinical examination and supporting photographs, skeletal evaluation with standardized radiographs, and dental evaluation with study dental casts.

Mandibular prognathism is a common clinical problem; however, its prevalence varies relative to the population. The highest incidence has been observed in the Asian population (15%), and the lowest has been observed in the Caucasian population (1%) [3]. Obwegeser [4] first demonstrated the possibility of repositioning the maxilla in a stable consistent manner and reported simultaneous repositioning of the maxilla and mandible. Most maxillofacial deformities can be managed with four basic osteotomies [5]: the maxilla with the Le Fort I-type osteotomy, the mandible with the sagittal split ramus osteotomy, the vertical ramus osteotomy, and the horizontal osteotomy of the chin.

Deformities of the chin can exist independently of mandibular deformities, and the chin can be abnormally proportioned without occlusal involvement. Horizontal osteotomy of the symphysis (osseous genioplasty) is a far more versatile procedure, in which the chin can be repositioned in multiple planes allowing for the correction of significant sagittal and vertical deformities of deficiency (microgenia) or excess (macrogenia), and this procedure is also used to correct facial asymmetry [6].

Currently, sagittal ramus osteotomy is the primary choice for correcting most cases of mandibular retrognathism and prognathism. The surgical option for extreme cases of mandibular prognathism is extraoral vertical ramus osteotomy (EVRO) or intraoral vertical ramus osteotomy (IVRO) or inverted L osteotomy [6]. The extraoral vertical ramus osteotomy procedure was the first one to be described and preferred in most instances.

EVRO is a very simple surgical procedure that takes less time than other mandibular procedures. The advantages of EVRO are simple osteotomy cuts and minimal complications. EVRO is a well-established technique for the correction of mandibular prognathism with the documented optional improvement of occlusion and facial esthetics as well as stability of the facial skeleton. Calderon et al. [7] characterized the extraoral approach as simple, allowing excellent visibility, and stated that the intraoral approach should be reserved for those who have the tendency to form keloid and those who objected to extraoral scars. Various benefits have been reported with orthognathic surgery including improved masticatory function, reduced temporomandibular joint pain, and improved facial esthetics. The purpose of this study is to evaluate the versatility of EVRO for mandibular prognathism.

## Materials and methods

Ten patients who were referred to the Department of Oral and Maxillofacial Surgery with the chief complaint of mandibular prognathism were enrolled in our prospective study. Patients with mandibular prognathism who were in the age group of 18-25 years, who had completed active growth, and who were motivated enough to comply with the treatment regime were included in the study. The completion of active growth was assessed by taking serial cephalograms for the last two years before the surgery and after superimposition of the cephalometric images; if no change was observed in the position of the cephalometric landmarks, then it was considered that the patient had completed active growth. Patients with mandibular retrognathism, a history of keloid tendency, and those who are medically compromised were excluded from the study. Ethical approval was obtained for the study from the institutional ethical committee, and informed consent was obtained from each patient in the regional language (Tamil) explaining the nature of the surgical procedure and the benefits and risks of participating in the study.

A thorough clinical examination was carried out on each individual. Routine investigation, model analysis, and radiographic analysis were done. A presurgical assessment was done to evaluate the general condition of the patient. Cephalometric analysis was done to assess the magnitude of mandibular excess in each individual. Due considerations were given to extreme cases of mandibular prognathism (i.e., more than 7 mm of setback). Presurgical orthodontic treatment was done to decompensate dental components.

**Figure 1:**
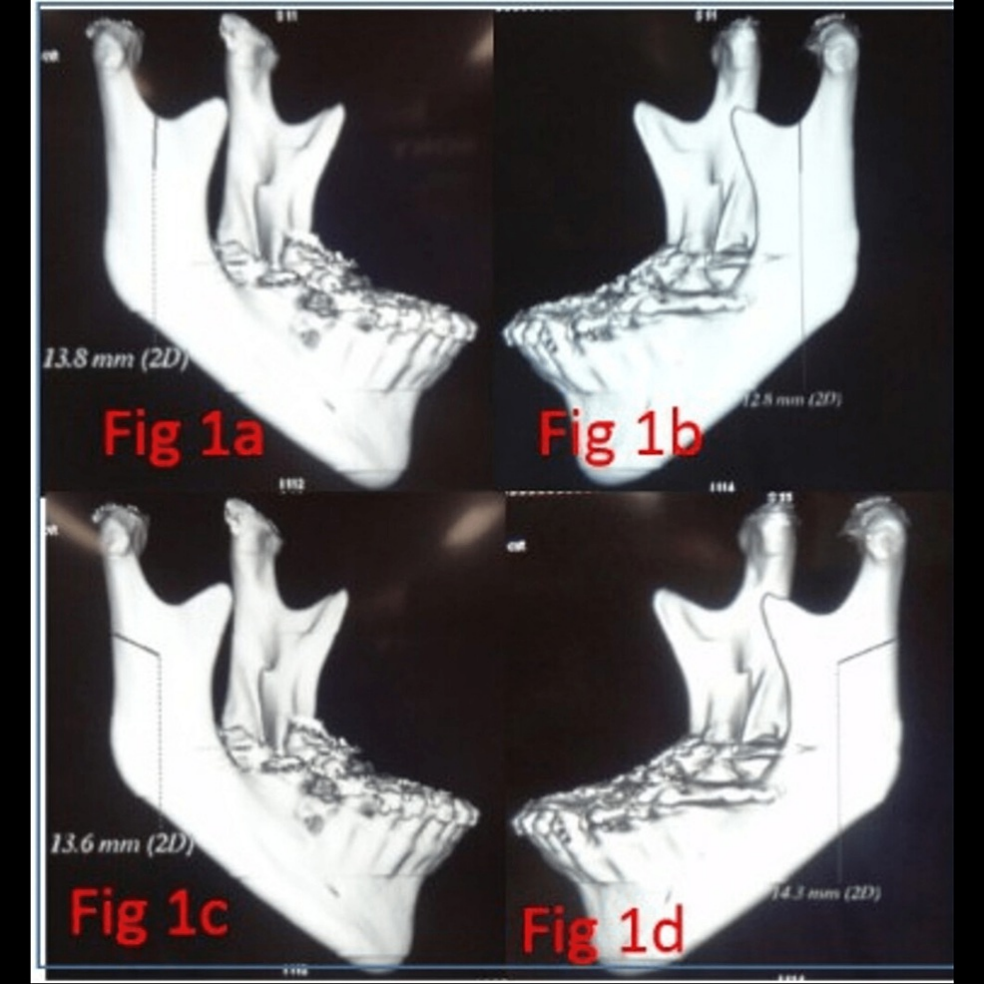
(a) Sigmoid notch to antilingula, (b) sigmoid notch to antilingula, (c) posterior border of the mandible to antilingula, and (d) posterior border of the mandible to antilingula

**Figure 2:**
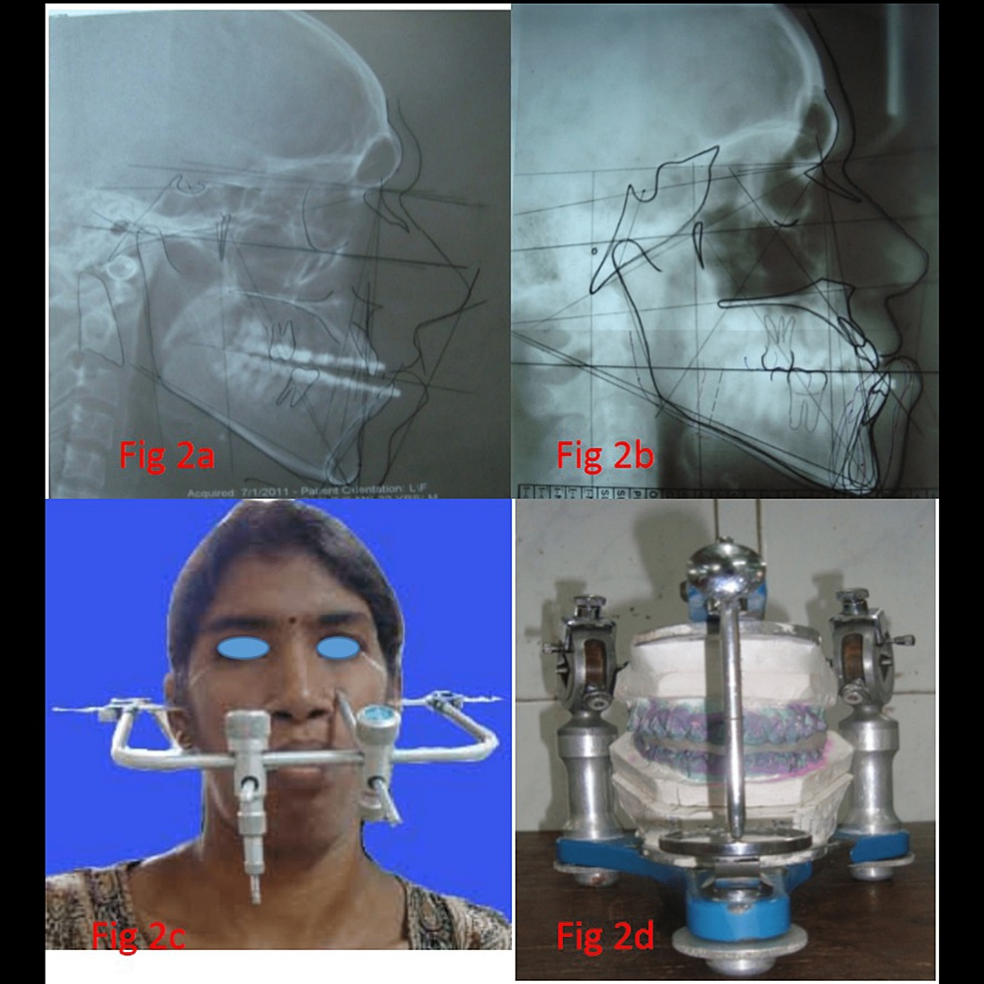
(a) Preoperative lateral cephalogram, (b) prediction tracing, (c) face bow surgery, and (d) model surgery and splint fabrication

A computed tomography scan analysis of the mandible was done. Antilingula on the buccal side and mandibular foramen on the lingual aspect were located, and the distance from the center of sigmoid notch to the antilingula and the mandibular foramen was measured (Figure 1, Panels a and b). Then the distance from the posterior border to the antilingula was also measured (Figure 1, Panels c and d). Taking this as a guide, an osteotomy cut in the ramus was made. Prediction tracing was done for each patient in the lateral cephalogram taken preoperatively followed by face bow transfer in a semi-adjustable anatomical articulator, and model surgery and splint fabrication were done (Figure 2, Panels a-d).

The parameters used to evaluate the outcome of the surgical procedure were time taken for the surgical procedure, facial harmony both in frontal and profile views postoperatively, intraoperative, and postoperative complications, and the assessment of the postoperative results was done through lateral cephalograms. The cephalometric measurements assessed preoperatively and postoperatively were posterior vertical height measured perpendicular to horizontal plane (HP) from posterior nasal spine to nasion (PNS-N perpendicular to HP), anterior nasal spine to posterior nasal spine (ANS_PNS), and the ramus height using articulare to gonion (Ar-Go). The reference planes used were Frankfort horizontal plane (FHP), Sella-Nasion plane (S-N), mandibular plane, and occlusal plane.

Surgical procedure

**Figure 3:**
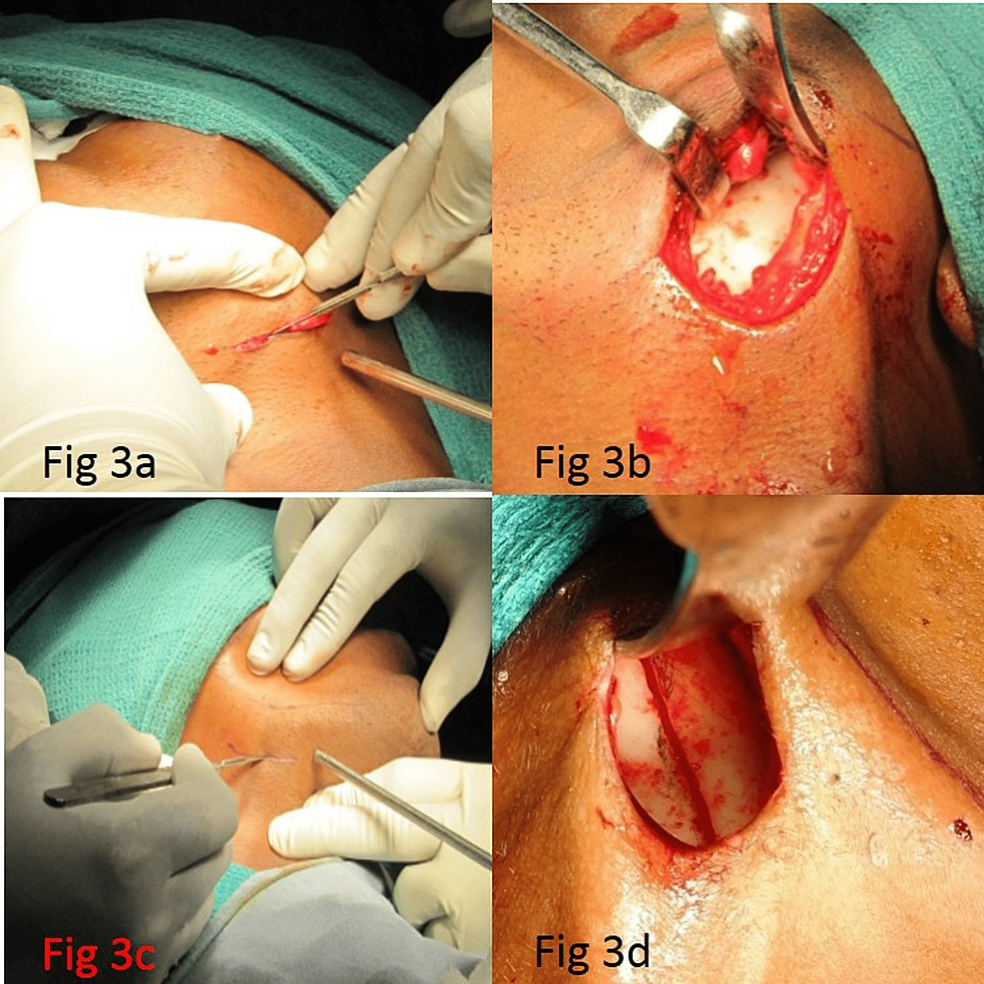
(a) Submandibular incision, (b) exposure of ramus, (c) submandibular incision, and (d) osteotomy cut

The EVRO was done under general anesthesia through nasoendotracheal intubation by the same single surgeon for all patients to avoid bias. Risdon’s [8] submandibular incision was placed in the submandibular region (Figure 3, Panel a and c), approximately 2 cm below the angle at the inferior border of the mandible. The incision was given with no. 15 blade, and the dissection was carried through the skin, subcutaneous tissue, platysma, and superficial layer of the cervical fascia. Care was taken to avoid injury to the marginal mandibular nerve. The facial vein and facial artery if encountered were clamped and ligated to achieve hemostasis, and the marginal mandibular nerve was identified and retracted posterosuperiorly and protected. A combination of sharp and blunt dissection was used at the inferior border of the mandible. After incision through the periosteum, the lateral surface of the ramus was exposed by stripping the masseter leaving some attachment in the proximal segment (Figure 3, Panel b).

After exposing the lateral surface of the ramus, the prominence of the antilingula was identified (Figure 1, Panel b). Computed tomography scan measurements made were used as a guide for making osteotomy cuts in the ramus. Vertical ramus osteotomy cut was made from the midpoint of the sigmoid notch, extending downward and posterior to the antilingula prominence down to the angle of the mandible (Figure 3, Panel d). The bony cut was made with 701 SS White Surgical Bur with a slight angulation of 110˚ to facilitate the overlapping of the proximal segments with the distal fragment. Adequate precaution was taken to maintain sufficient width of the proximal segment while making vertical ramus osteotomy so that the blood supply was maintained to the temporomandibular joint (TMJ) capsule and attachment of lateral pterygoid muscle superiorly. Care was also taken to avoid damage to the inferior alveolar nerve by making the osteotomy cut posterior to the nerve canal.

**Figure 4:**
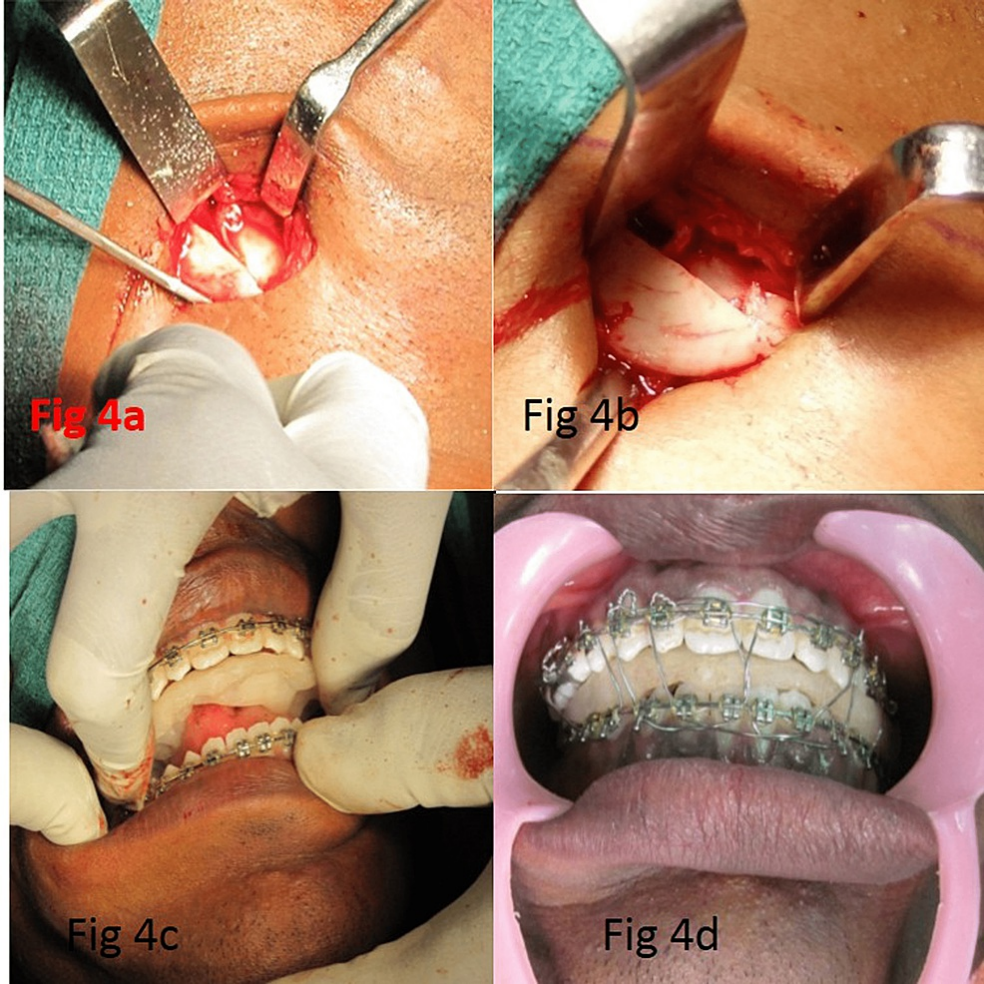
(a) Overlapping segments, (b) overlapping segments, (c) mandibular setback with splint, and (d) IMF with splint IMF: Intermaxillary fixation.

The osteotomy cut was completed using a thin osteotome, and the proximal fragment was gently mobilized by stripping a small portion of the periosteum and muscle at its lower end on the lateral aspect. The medial pterygoid muscle was gently detached on the medial aspect of the proximal segment. This segment was positioned lateral to the major distal fragment. An identical procedure was performed on the other side of the mandible to complete the EVRO bilaterally. The distal segment was pushed back to the desired level (Figure 4, Panel a and b).

After completion of the osteotomy, the mobility of the segments was confirmed intraorally, the mandible was placed in the predetermined position in relation to the maxilla with the help of a surgical splint (Figure 4, Panel c), and the segment was immobilized using the orthodontic bands with 26-gauge stainless steel wires (Figure 4, Panel d).

The surgical wound was closed in layers after obtaining sufficient hemostasis. Care was taken to ensure that the proximal segment overlaps the distal segment at the time of closure. The patient was kept under intermaxillary fixation (IMF) for a period of seven to 10 days after surgery. Light elastic traction was used to guide the occlusion for the next four to five weeks while initial bone healing occurred. All the patients were subjected to nasal feeding through Ryle’s tube for 10 days, followed by liquid food through oral feeding till the IMF was released.

Statistical analysis

Data was collected and subjected to statistical analysis using SPSS software version 27.0 (IBM Corp., Armonk, NY). A paired sample t-test was carried out to compare the preoperative and postoperative cephalometric evaluation.

## Results

Ten patients (seven male and three female patients) with chief complaints of mandibular prognathism were included in the study. The mean age of the study participants (N = 10) was found to be 20 ± 2.263 years. There was a reverse overjet of 7 mm in eight patients and 8 mm in two patients. The average mandibular setback achieved was 10 mm (Table 1).

**Table 1:** Preoperative and intraoperative evaluation

S. No	Age/Sex	Diagnosis	Reverse Overjet	Total Time Taken for Surgery in Minutes	Surgical Access	Visibility
1.	20/M	Mandibular prognathism	-7 mm	94	Excellent	Excellent
2.	22/M	Mandibular prognathism	-7 mm	80	Excellent	Excellent
3.	18/F	Mandibular prognathism	-7 mm	87	Excellent	Excellent
4.	18/F	Mandibular prognathism	-8 mm	80	Excellent	Excellent
5.	20/M	Mandibular prognathism	-8 mm	84	Excellent	Excellent
6.	21/M	Mandibular prognathism	-7 mm	100	Excellent	Excellent
7.	25/M	Mandibular prognathism	-7 mm	88	Excellent	Excellent
8.	22/M	Mandibular prognathism	-7 mm	90	Excellent	Excellent
9.	18/M	Mandibular prognathism	-7 mm	102	Excellent	Excellent
10.	19/M	Mandibular prognathism	-7 mm	104	Excellent	Excellent

**Figure 5:**
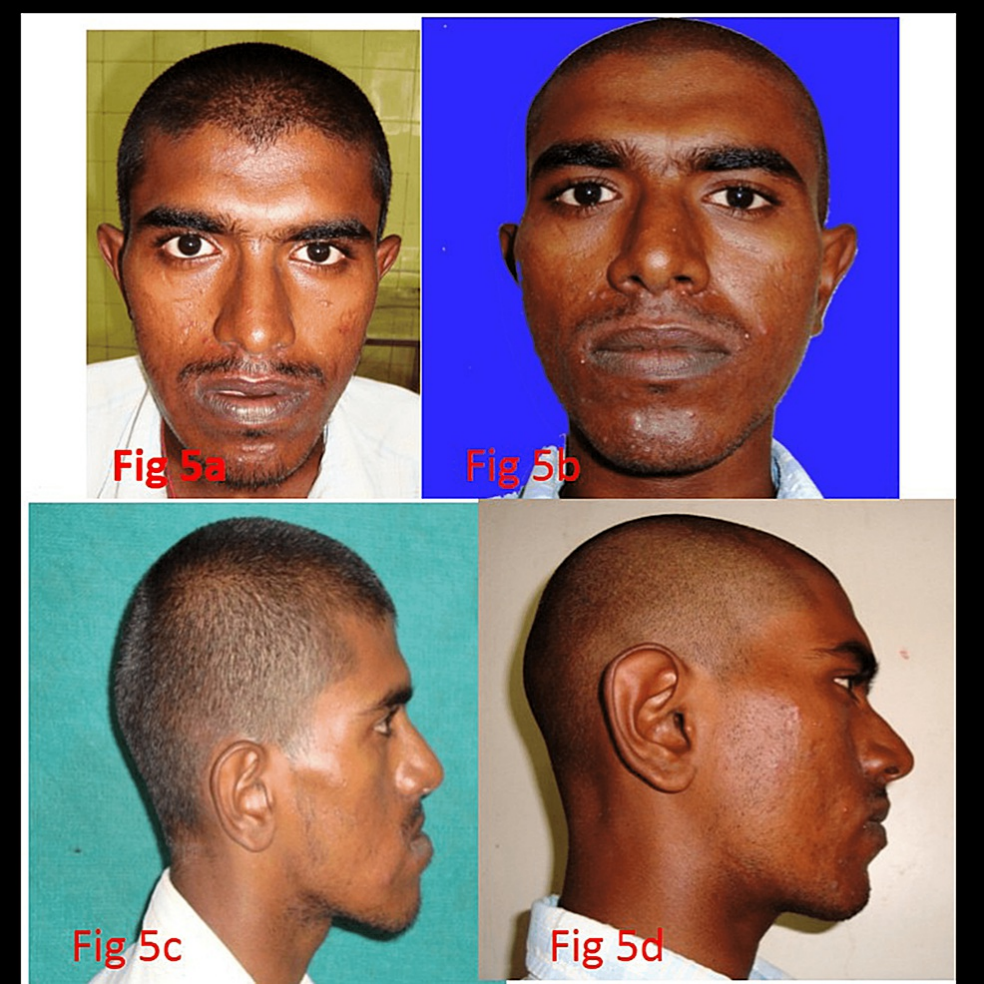
(a) Preoperative frontal view, (b) postoperative frontal view, (c) preoperative profile view, and (d) postoperative profile view

The time taken for the entire surgical procedure, starting from incision to closure, varied between 80 and 94 minutes with an average time taken for the surgery of about 90 ± 8.80 minutes. There was a gradual reduction in the operating time for the successive cases. We had excellent visibility and access to the surgical site for all the patients. Immediate postoperative results showed clinically satisfying frontal and profile appearances (Figure 5, Panels a-d).

**Figure 6:**
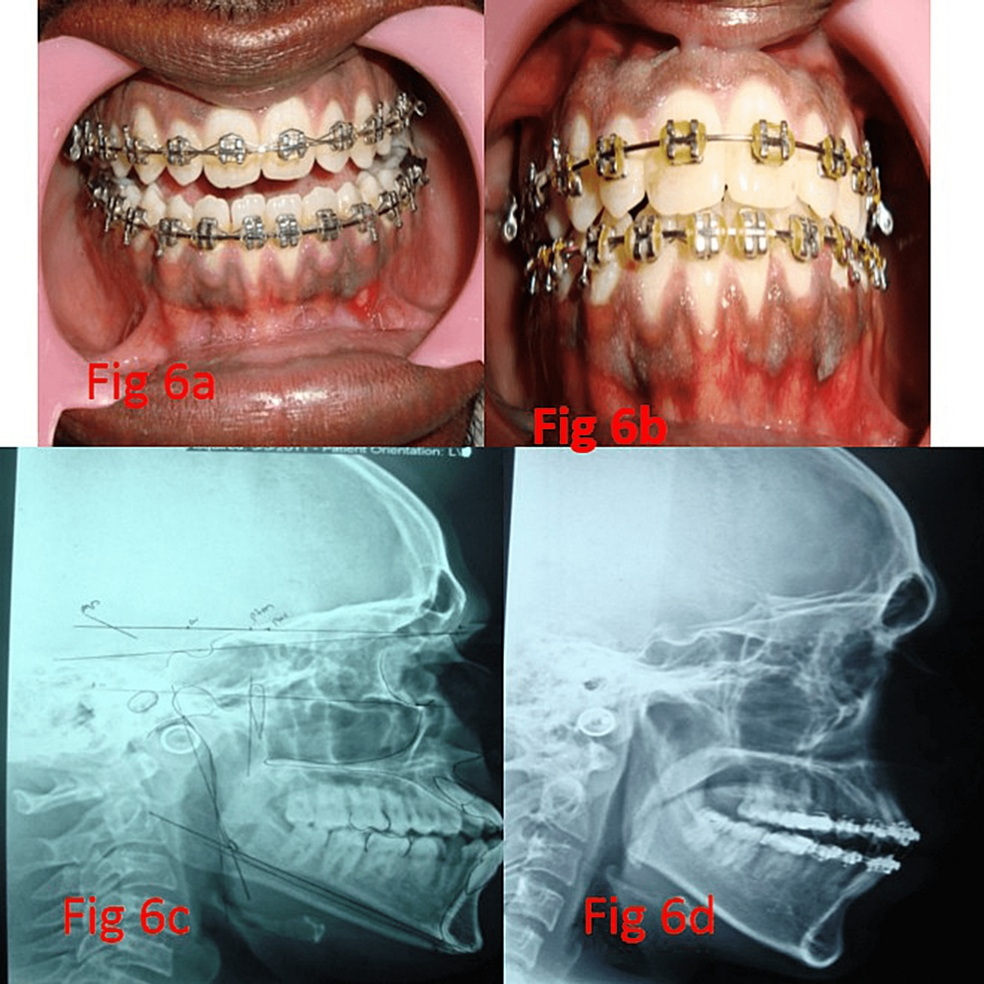
(a) Preoperative occlusion, (b) postoperative occlusion, (c) preoperative lateral cephalogram, and (d) postoperative lateral cephalogram

A class I molar occlusion was achieved in all patients (Figure 6, Panels a and b). It was found that there was a statistically significant difference between preoperative (mean = 53.4, SD = 5.854) and postoperative evaluation (mean = 47.5, SD = 5.039) of PNS-N N perpendicular to HP (mm) with p < 0.001. Similarly, there was a statistically significant difference between preoperative (mean = 81.4, SD = 2.716) and postoperative evaluation (mean = 74.4, SD = 3.627) of mandible body length (mm) with p < 0.001.

However, no statistically significant difference exists between the preoperative and postoperative evaluation of ANS_PNS (mm) and Ramus height Ar-Go (mm) (Table 2). Esthetically satisfying results were observed in all the patients during the follow-up period, both clinically and radiographically (Figure 6, Panels c and d). The concave profile in the preoperative period transformed into a straight profile.

**Table 2:** Cephalometric analysis: preop and postop PNS-N: Posterior nasal spine to nasion; HP: Horizontal plane; ANS_PNS: Anterior nasal spine to posterior nasal spine; Ar-Go: Articulare to gonion.

S. No	PNS-N 1 HP in mm		ANS_PNS in mm		Ramus Height Ar-Go in mm		Mandible Body Length in mm	
	Preop	Postop	Preop	Postop	Preop	Postop	Preop	Postop
1.	58	52	60	60	49	49	86	81
2.	43	41	51	51	49	48	78	71
3.	54	50	54	54	59	59	82	77
4.	53	46	53	53	57	56	84	78
5.	48	41	53	53	67	67	80	72
6.	57	51	54	54	56	56	82	74
7.	59	52	58	58	58	58	81	72
8.	46	40	60	60	49	49	79	72
9.	56	50	52	52	48	48	78	70
10.	60	52	55	55	57	57	84	77

No significant intraoperative complications were observed in this study, except for one patient who had nausea. The IMF was disrupted and the orthodontic brackets became loose due to the violent behavior of one patient, which led to the supra-eruption of the lower anterior teeth. This was corrected by applying IMF with Ivy loop eyelet wiring. There was no edema or hematoma in any of our patients in the follow-up period (Table 3).

**Table 3:** Postoperative evaluation TMJ: Temporomandibular joint; IMF: Intermaxillary fixation.

S. No	Pain	Fever	Infection	Hospital Stay	Nerve Injury	Other Complications	TMJ Problems	Stability After Release of IMF	Clinical Profile	CT Obliteration of Sigmoid Notch
1.	Nil	Nil	Nil	5 days	Nil	Nil	Nil	Stable	Straight	Obliterated
2.	Nil	Nil	Nil	5 days	Nil			Stable	Straight	Obliterated
3.	Nil	Nil	Nil	5 days	Nil	Nil	Nil	Stable	Straight	Obliterated
4.	Nil	Nil	Nil	6 days	Nil			Stable	Straight	Obliterated
5.	Nil	Mild for one day	Nil	5 days	Nil	Nil	Nil	Mild relapse in the dental component	Straight	Obliterated
6.	Nil	Nil	Nil	6 days	Transient marginal mandibular nerve palsy for 3 weeks			Stable	Straight	Obliterated
7.	Nil	Nil	Nil	5 days	Nil	Nil	Nil	Stable	Straight	Obliterated
8.	Nil	Nil	Nil	6 days	Nil			Stable	Straight	Obliterated
9.	Nil	Nil	Nil	5 days	Nil	Nil	Nil	Stable	Straight	Obliterated
10.	Nil	Nil	Nil	5 days	Nil			Stable	Straight	Obliterated

The hospital stay of our patients ranged from seven to 10 days, which includes the preoperative stay also. One patient had a slight deviation of the lower lip to the left side in the postoperative period, probably due to retraction, and weakness of the marginal mandibular nerve would have occurred on the right side. The deviation of the lower lip resolved in two to three weeks, and the patient apparently became normal. After removing IMF (after six weeks), one patient had a mild relapse and developed an edge-to-edge incisor relation during the third-month follow-up. Further relapse was not noticed in the same patient, suggesting good stability fairly once the postsurgical orthodontic settling was started. The mean follow-up period of all the patients was three years.

## Discussion

Dentofacial deformity is apparent in the majority of the population, of which mandibular prognathism contributes to the maximum, and a substantial amount of the population is found to suffer from this skeletal deformity. The incidence of mandibular prognathism is significantly higher than that of other craniofacial deformities [9]. However, its prevalence varies from place to place and race to race. The highest incidence of 15% was observed in the Asian population, and the lowest incidence of 1% was observed in the Caucasian population [10].

Bilateral sagittal split ramus osteotomy was the most widely used surgical procedure for the correction of mandibular retrognathism and prognathism. For most extreme cases of mandibular prognathism, “EVRO” or “IVRO” is the treatment of choice [11]. EVRO was first introduced by Caldwell and Letterman in 1954 [11]. It was first reported by Robinson in 1956 and Hinds in 1957 [12].

Vertical subcondylar osteotomies of the mandibular rami for correction of mandibular prognathism may be performed through an extraoral or intraoral approach. The ease of approach, access, and surgical visibility have made many surgeons prefer “EVRO” for the correction of mandibular prognathism [13]. Because of the external scar and the chances for damage to the facial nerve, many authors preferred the intraoral approach [14]. In this study, we have not come across such an incidence of conspicuous scar or facial nerve damage except in one case where there was a weakness of the marginal mandibular nerve, which resolved in three weeks. Öhrnell et al. reported a transient hypersensitization of the inferior alveolar nerve in 19.2% of the patients and no complaints of scars in their study on EVRO with internal fixation [15]. Hågensli et al. [16] recorded neurosensory disturbance in one out of 65 patients in their extraoral vertical subcondylar osteotomy with a rigid fixation for prognathic mandibles. This result was attributed to a different osteotomy design and usage of monocortical osteosynthesis plates, thereby avoiding interference with the mandibular foramen. Peleg et al. [17] reported neurosensory disturbances of the inferior alveolar nerve in 11.54% of patients who had undergone sagittal osteotomy and 5.08% of patients who underwent IVRO. The results of the current study and other studies suggest that ramus osteotomies had fewer chances of nerve injury.

The operation time taken for “EVRO” in this study varied between 80 minutes to 94 minutes with a mean of 90 ± 8.80 minutes as compared to the study conducted by Tornes and Gilhuus-Moe [13] in which the operation time for “EVRO” ranged from 50 to 180 minutes with a mean of 88 minutes. In contrast, Öhrnell et al. [15] reported the duration of the surgical procedure as two hours and 26 minutes, whereas Peleg et al. reported that the duration was two hours and seven minutes with IVRO, thereby indicating that extaoral ramus osteotomy is faster than intraoral ramus osteotomy. Prophylactic antibiotics were given to all the patients to avoid contamination of an extraoral wound with intraoral manipulation of the segments. None of our patients received blood transfusion intraoperatively or postoperatively since the blood loss recorded was less than 500 ml in all the cases. Wang and Waite [18] estimated the blood loss for the “EVRO” technique to be between 50 and 650 ml with a mean of 180 ml, and Öhrnell et al. estimated the blood loss to be 76 ml [15].

The cephalometric changes observed through lateral cephalograms showed a significant difference in the setback of the mandible, and the long-term stability of the mandible was satisfactory both clinically and functionally, similar to the study of long-term stability of the EVRO and IVRO groups by Nordin et al. [19]. They concluded that the final choice of a surgical approach should be made mainly with regard to the clinical aspects. Li et al. [20] compared the postoperative stability of sagittal split ramus osteotomy and IVRO and concluded that the horizontal stability at the B-point was superior in the IVRO group. Nihara et al. [21] studied the short-term and long-term postoperative skeletal changes following IVRO from lateral cephalograms. They observed a clockwise rotation of the distal segment of the mandible in the short term, which could be due to the adaptation of the masticatory system. In the long term, the menton had moved anteriorly only by .9 mm, and the relapse ratio was just 15.3%, thereby substantiating the excellent long-term stability of the ramus osteotomies. In our study among 10 patients of mandibular prognathism, nine patients underwent presurgical orthodontics, and presurgical orthodontics was not done for one patient (90%) similar to the study conducted by Tornes and Gilhuus-Moe [13] in which 86% of the patients had preoperative orthodontics. However, the stability of the procedure depends on the amount of retrusion, the rigidity of the fixation, and the continuous growth of the mandible [17].

IMF was done using the orthodontic appliances in nine patients and Ivy loop eyelet wiring in one patient. In one case, supra-eruption of the lower incisor was observed during the IMF period, and the pressure on that tooth was relieved to avoid further complications. A review of the literature reveals that many authors preferred IMF with the existing orthodontic appliances [22]. Mobarak et al. [23] compared the stability of EVRO using two methods of fixation, namely plates versus maxillomandibular fixation and skeletal suspension. They reported that the maxillomandibular fixation group demonstrated the posterior movement of the mandible with an increase in mandibular plane angle, shortening of the rami, and dental compensation, thereby resulting in a small anterior relapse upon release of the maxillomandibular fixation and skeletal suspension, whereas this was not observed in the plate fixation as only 10% anterior relapse tendency was recorded, thereby suggesting that plate fixation in EVRO provided excellent long-term stability. This anterior relapse tendency was observed in four of our patients as we had not performed rigid fixation. All our patients had speech and feeding-related inconveniences because of the maxillomandibular fixation, which seems to be the major drawback of the extraoral ramus osteotomy [24].

Despite the fact that all patients received anti-emetic treatment in our study among 10 patients, only one had nausea (20%) when compared to the study conducted by Malekzadeh et al. [15] in which 21.2% of the patients had nausea. Mild to moderate edema was seen in all our patients till the fifth postoperative day patients. There was a gradual disappearance of the edema since the patients were under corticosteroid therapy for five days. Infection was not experienced in any of our cases during the follow-up period as observed by Tornes [13] in his study on the “EVRO” group.

The total period of hospitalization ranged from seven to 10 days with a mean of 5.2 days as compared to the study conducted by Tornes and Gilhuus-Moe [13] in which he found that EVRO procedure needs less period of hospitalization i.e., three to 10 days with a mean of 4.8 days as compared to IVRO in which the period of hospitalization was three to 11 days. There was no incidence of parotid fistula in our cases since extreme precautions were taken during surgery to minimize the postoperative complications.

Hines et al. [25] revealed that EVRO was simple, allowing excellent visibility and access, and intraoral vertical ramus osteotomy is reserved for keloid formers and patients who objected to extraoral scars. The EVRO carried out in all our cases has provided excellent exposure and good access. Even though the surgical procedure produced a scar, it was not conspicuous, and keloid was not observed in any of our patients. No patients complained of a scar problem.

Tornes and Gilhuus-Moe [13] in his study stated that patients with very high rami were considered technically difficult in IVRO. These patients were operated on with the extraoral vertical ramus osteotomy technique. All our cases had a very high ramus and were subjected to EVRO. From the observation made in our study, “EVRO” is a well-established technique for the correction of mandibular prognathism with the documented optional improvement of occlusion and facial esthetics as well as the stability of the established skeletal relationship.

This study has a few limitations. Virtual surgical planning was not done in our study. In a recent meta-analysis comparing the effectiveness of traditional (TSP) and virtual surgical planning (VSP) for orthognathic surgery, VSP and patient-specific osteosynthesis were found to be significantly better in predicting certain reference areas, in addition to the advantage of reducing the surgical times, even for inexperienced surgeons. Patient-specific cutting guides and osteosynthesis mean less risk for intraoperative bad splits and neural injuries, while a shorter operative time means less blood loss, less anesthesia time, and lower surgical risk [26]. A three-dimensional evaluation of the surgical outcomes has not been done. The postoperative results were analyzed only using lateral cephalograms. A further limitation of this study is the lack of a control group. Moreover, the sample size is very small. Hence, future research should be directed toward a three-dimensional comprehensive analysis of the surgical outcomes [27], and a comparative study comparing EVRO with IVRO or bilateral sagittal split osteotomy with a larger sample size is needed to ascertain the supremacy of this approach.

## Conclusions

EVRO is an acceptable surgical procedure due to its ease of execution, lack of complications, and positive outcomes. Due to the lack of relapses and the almost inconspicuous scar, it is observed that this surgical procedure offers the best chance for long-term results, provided that extreme care is taken in the selection of cases (mandibular prognathism of >7 mm). In the future, this technique will be valuable in the hands of maxillofacial surgeons when it is preceded by a thorough workup and careful patient selection.
